# Sézary Syndrome—Eosinophilia Explained by a Blood Film

**DOI:** 10.1002/ajh.27673

**Published:** 2025-03-26

**Authors:** Merel T. A. Soeterik, Joris Janssen, Leo M. Budel, Anton W. Langerak, Yorick Sandberg, Barbara J. Bain

**Affiliations:** ^1^ Department of Internal Medicine Maasstad Hospital Rotterdam the Netherlands; ^2^ Department of Pathology Maasstad Hospital Rotterdam the Netherlands; ^3^ Department of Immunology, Erasmus MC University Medical Center Rotterdam the Netherlands; ^4^ Centre for Haematology, St Mary's Hospital Campus of Imperial College Faculty of Medicine, St Mary's Hospital London UK

**Keywords:** eosinophilia, Sézary syndrome

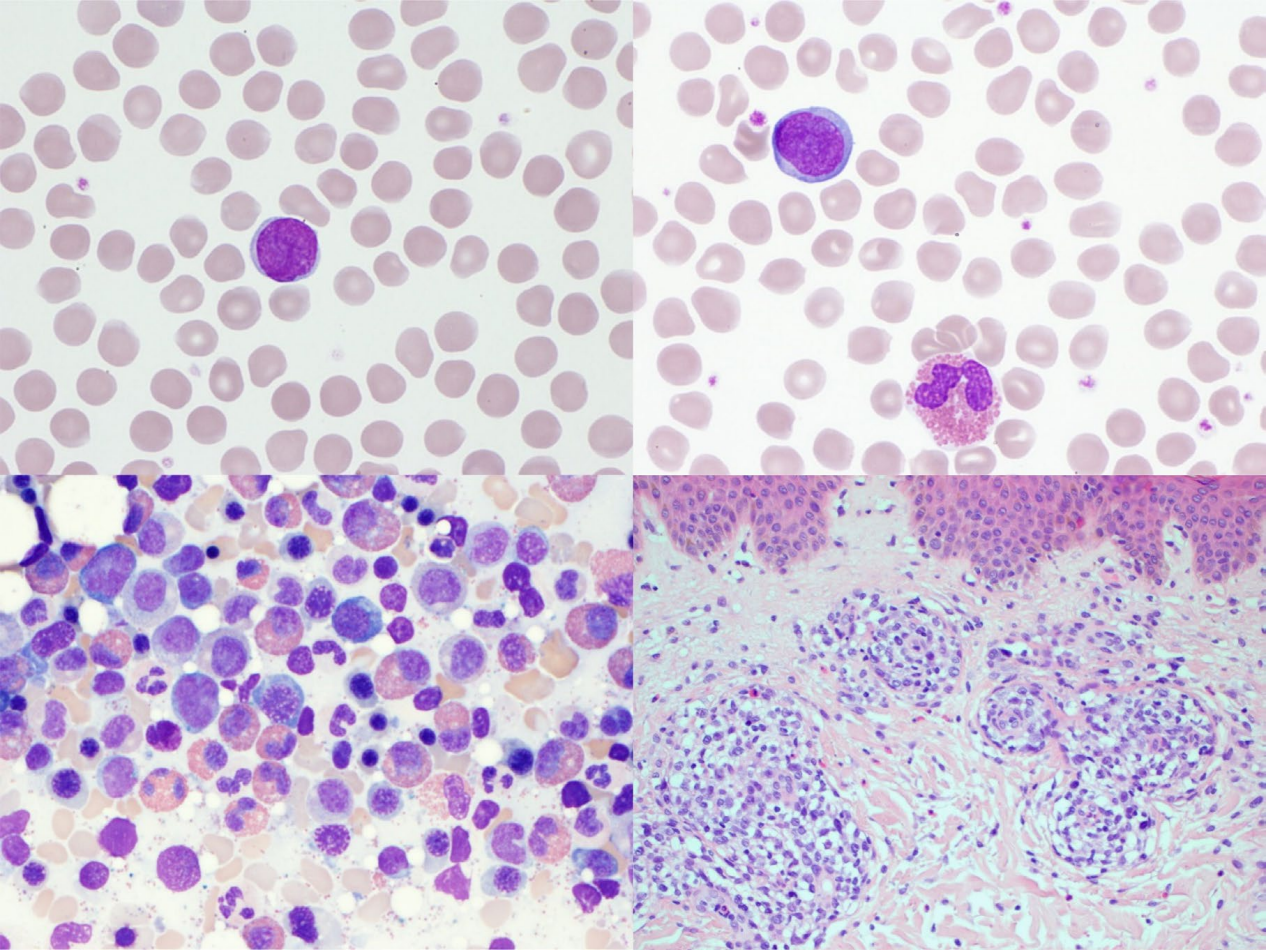

A 61‐year‐old man, with no significant medical history, presented with a six‐month history of fatigue, pruritus, night sweats, and weight loss. Physical examination revealed erythroderma and generalized lymphadenopathy. Laboratory tests showed prominent eosinophilia (white blood cell count 10.6 × 10^9^/L, eosinophils 2.9 × 10^9^/L). There was no lymphocytosis (lymphocyte count 1.1 × 10^9^/L). Lactate dehydrogenase (LDH) was elevated at 469 U/L. A peripheral blood smear (top left and top right, May–Grünwald–Giemsa [MGG], ×100 objective) highlighted marked eosinophilia alongside large, atypical lymphocytes with convoluted and grooved nuclei. Lymph node biopsy confirmed infiltration by an aberrant T‐cell population. Bone marrow examination showed atypical large T cells accounting for 14% of the total cell population and an eosinophil fraction of 25% (bottom left, MGG, ×100). A skin punch biopsy (bottom right, hematoxylin and eosin, ×20) demonstrated superficial perivascular lymphocytic and eosinophilic dermatitis, with no spongiosis or epidermotropism. Immunohistochemical analysis identified abnormal lymphocytes expressing CD4 with a CD4:CD8 ratio ≥ 10 and concurrent loss of CD5 and partial loss of CD7. Subsequent immunophenotyping of peripheral blood, bone marrow, and lymph nodes confirmed the presence of an identical clonal T‐cell population with loss of CD26 expression also being shown.

Based on these peripheral blood and histopathological findings in the context of erythroderma, the diagnosis of Sézary syndrome was established. Sézary syndrome is an aggressive form of mature T‐cell leukemia, characterized by refractory symptoms and a poor prognosis. Eosinophilia occurs frequently [1] and is believed to result from the secretion of specific Th2‐associated cytokines by neoplastic cells [2].

There are innumerable causes of eosinophilia. Sézary syndrome is one of a number of causes that may be revealed by peripheral blood examination. This may be so even when the lymphocyte count is not increased. In addition, the eosinophil count may be of prognostic significance in cutaneous T‐cell lymphomas [1].

## Conflicts of Interest

The authors declare no conflicts of interest.
